# Acute pancreatitis secondary to hyperlipidemia in an 11-year-old girl: A case report and review of literature

**Published:** 2013-03-25

**Authors:** NR Bălănescu, L Topor, A Ulici, FB Djendov

**Affiliations:** Department of Pediatric Surgery, “Grigore Alexandrescu" Clinical Emergency Hospital for Children, Bucharest, Romania, “Carol Davila" University of Medicine and Pharmacy, Bucharest, Romania

**Keywords:** acute pancreatitis, hyperlipidemia, hypertriglyceridemia, acute surgical abdomen, abdominal ultrasonography

## Abstract

We report the case of an 11-year-old female with acute pancreatitis, and review the literature highlighting the presenting symptoms and signs, laboratory tests, and investigational tools that helped in the establishment of a correct diagnosis. First, the patient presented to a regional hospital reporting abdominal pain, vomiting and liquid stool. She was admitted with the diagnosis of acute surgical abdomen and underwent surgery. Upon admission in our department, laboratory findings showed high values for total lipids = 2600 mg/dL and triglycerides 1200 mg/dL. Abdominal ultrasound revealed a pancreas with a small enlargement of the head (19 mm), and with a heterogeneous structure of the parenchyma. Abdominal computed tomography showed small left pleural collection and a high-dimensioned pancreas, particularly at the head, with heterogeneous structure, and peripancreatic collections. The patient was treated by fasting, gastric decompression by nasogastric tube, and intravenous antibiotherapy followed by antialgic and antispasticity treatment. Time of the patient’s first feeding was after the 7th day of hospitalization. The patient was discharged in a very good condition after 22 days of hospitalization.

## Introduction

The incidence of pediatric pancreatitis has increased significantly in the past two decades. It is estimated that 2 to 13 new cases occur annually per 100,000 children [**1**]. Pancreatitis affects a heterogeneous population of children, and symptoms range from mild to life – threatening [**2**]. In acute pancreatitis, although the pathophysiology and functional consequences in children are identical to those observed in adults, its etiology differs significantly. The most common causes of pancreatitis in children include 1) systemic diseases, such as systemic lupus erythematosus, Henoch – Schönlein purpura, Kawasaki disease, Crohn’s disease, hyperlipoproteinemia, and hypertriglyceridemia (rarely); 2) different drugs and toxins, such as thiazides, furosemide, cimetidine, estrogen, and tetracycline; 3) infections; 4) obstructive diseases; 5) trauma; and 6) hereditary pancreatitis. 

 The association between hyperlipidemia and acute pancreatitis was first noted by Speck in 1865 [**3**], and since then, the interrelationship has been studied by many researchers [**4-6**], most importantly by Cameron and associates [**7**]. Pancreatitis secondary to HTG typically presents as an episode of acute pancreatitis or recurrent acute pancreatitis, its chronic form is rare [**8**]. Clinical indicators of acute pancreatitis include the sudden onset of a constant and sharp abdominal pain associated with nausea, vomiting and fever. Additionally, the presence of hyperamylasemia accompanied by a compatible clinical presentation is also suggestive of acute pancreatitis [**9**]. Diagnostic accuracy is further improved if there is an associated elevated lipase concentration (99% specificity). Serum lipase levels may remain elevated when the levels of amylase return to normal [**10**]. 

 Treatment of pancreatitis can depend on several factors. First, acute pancreatitis is usually associated with a seric level of triglycerides above 1000 mg/dL, a factor that can be used to determine the necessary therapy [**11**]. Acute pancreatitis can be treated with fasting, gastric decompression by nasogastric tube, and analgesia with meperidine and IV metamizole magnesium. Fluid therapy can also be initiated as well as intravenous bicarbonate. In addition, continuous insulin perfusion can be used for diabetic ketoacidosis treatment and to control the extremely high triglyceride levels [**12,13**]. In acute pancreatitis, the exact cause is the main factor that determines which, if any, surgical procedure should be performed. Thus, operative interventions can vary from ERCP (endoscopic retrograde cholangiopancreatography) with sphincterotomy and balloon sweeping of choledochus to aggressive percutaneous drainage of pancreatic fluid and necrosectomy, to laparotomy and laparoscopy [**14**].

## Case report

The patient is an 11-year-old girl who presented to a regional hospital with abdominal pain, vomiting and liquid stools. At that time, she was given antialgic treatment and then discharged. Because the abdominal pain persisted, she returned to the hospital and was admitted with the diagnosis of acute surgical abdomen. Considered an emergency diagnosis, the patient underwent an appendectomy under general anesthesia with peritoneal drainage. Necrotic hemorrhagic pancreas and mesenteric abscess were discovered intraoperatively, and consequently the patient was transferred to the Pediatric Surgery Department of "Grigore Alexandrescu" Clinical Emergency Hospital for Children. 

The patient arrived in our unit conscious and cooperative, but displaying fever along with a highly worsened status and characteristic facial signs of suffering. She also had a nasogastric suction tube with approximately 100 ml of bilious emesis. The patient was breathing efficiently with an easy dyspnea, a respiratory rate of 18 breaths/minute, an oxygen saturation level above 90 % and a pulse of 86 bpm. Her abdomen was diffusely and mildly tender to palpation, and she presented with multiple impaired drainage tubes (locations: splenic, hepatic, Douglas sack, sub mesenteric). Diuresis was present as observed through the urinary catheter with hyperchromic urine. 

Imaging exams were performed, and, on the pulmonary Rx, we were able to distinguish the nasogastric tube present in the esophagus and moderate accentuation of the moderate perihilar interstitial bronchial image. 

The patient underwent contrast-enhanced CT imaging upon admission, which revealed left pleural collection in a small quantity with a minimum left compression atelectasis, and a high-dimensioned pancreas, particularly at the head, with heterogeneous structure and peripancreatic collections. Similar fluid collections could also be observed between the liver and kidney, between the spleen and kidney and both sides of the perirenal. The liver, spleen, and kidneys were all normal.

**Fig. 1:**
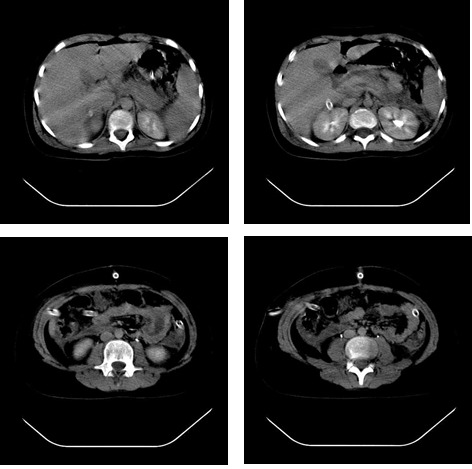
Important aspects of CT scan at admission in our unit

Abdominal ultrasonography (US) was also performed upon admission. The findings revealed 1) an enlarged gallbladder that was 7-cm-heigh, with of a transverse diameter of 3 cm and without calculi; 2) a pancreas with a small enlargement of the head (19 mm), a body of 15 mm and a tail of 17 mm, with a heterogeneous structure without images, that indicate collections and without dilatations of the Wirsung duct; and 3) moderate quantity of liquid in Douglas sack.

**Fig. 2:**
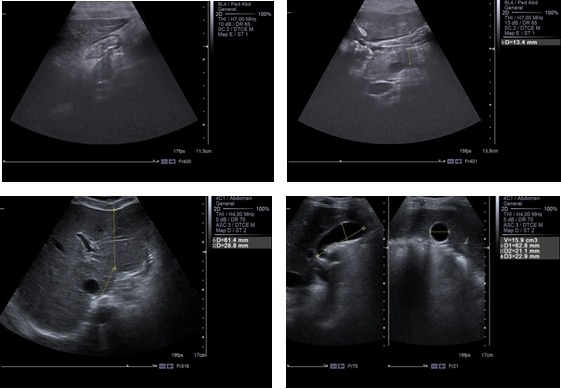
Important aspects of abdominal ultrasonography at admission in our unit

After 4 days of hospitalization, the patient underwent another abdominal CT scan, which showed no significant changes to the abdominal lesions detected during the first CT scan.

 After 20 days of hospitalization, abdominal ultrasonography was performed again. The findings noted some improvements including a pancreas with a smaller head enlargement (17 mm), a body of 14 mm and a tail of 15 mm having identical structure, and a smaller quantity of liquid in the Douglas sack. Therefore, a favorable outcome had occurred.

In terms of the laboratory tests, the patient presented with the following: WBCs = 3.9 *1000/µL (LY = 2,2%; Gran = 35,8% ), HGB = 11,5 g/dL, HCT = 33,4%, and PLT=255*1000/µL. Biochemistry tests showed values smaller than normal for total proteins = 5,45 g/dL and gamma GT = 12 IU/L, and greater values for total lipids = 2600 mg/dL, triglycerides = 1200 mg/dL, serum amylase = 86,04 mg/dL, cholesterol = 226,37 mg/dL and LDH = 572.07 IU/L.

 Compared with the admission labs, the findings at discharge differed as it follows: higher total proteins = 6, 73 g/dL, lower total lipids = 877, 75 mg/dL and lower cholesterol = 191 mg/dL, much lower triglycerides = 358, 40 mg/dL along with higher values for serum amylase = 102.29 mg/dL. 

 Other diagnostic tests included a urine culture that was positive for E. coli (>100.000 CFU/ml) with a antibiogram sensitive for ampicillin and augmentin, and an anti-HIV – antibodies screening, findings from which were negative. 

 During hospitalization, the patient maintained nasogastric intubation for gastric decompression. Additional treatments included fasting, intravenous antibiotherapy with Meronem, Ciprofloxacin, and Zyvoxid, and antialgic treatment consisting of Perfalgan, Algocalmin, and Chirocaine through epidural catheterization. Losec was administered to reduce the gastric acid secretion, which stimulates pancreatic secretion. Hypovolemic equilibration of the patient was also established. 

 The patient resumed oral feedings within several days after cessation of abdominal pain, nausea, vomiting and abdominal distention. The first resumption of oral feeding occurred on the 7th day after admission. The patient’s diet initially consisted of clear liquids (i.e., water, tea and soup) and was then advanced sequentially to soft solids (i.e., degreased yogurt or milk) and the limited intake of small amounts of kcal/d that were gradually increased as tolerated. Every meal was followed by Kreon administration. 

 The patient was discharged after 22 days of hospitalization with good status. Examination revealed a non-painful depressible abdomen, normal intestinal transit, normal diuresis, feeding regime and healing surgical wound.

## Discussion

Pancreatitis has been identified as the most common pancreatic disorder in children in recent reviews, such as those of Synn et al. [**15**] and Vane et al. [**16**]. Hypertriglyceridemia is a rare cause, contributing to acute pancreatitis in up to 7% of cases [**17**]. Our patient developed acute pancreatitis secondary to hyperlipidemia, a rare cause that can frequently lead to misdiagnoses. However, the diagnosis of pancreatitis can usually be made with reasonable certainty based on clinical, radiographic, and laboratory findings [**18**]. In our case, the clinical presentation included abdominal pain, vomiting and liquid stools. Further diagnostic evaluations included an abdominal US, simple computed tomography and a contrast-enhanced CT scan. The examination of our patient’s imaging results along with the data from the reviewed literature revealed dilatation of the intrahepatic and extrahepatic biliary ducts as well as an enlargement of the pancreas, being the primary echographic signs of pancreatitis in children [**19**]. Abdominal US has been shown to have 80% accuracy in the evaluation of pancreatitis, usually demonstrating decreased echogenicity of the pancreas [**15**]. Because of its accuracy, non-invasiveness, speed, portability, and relative inexpensiveness, US should be performed in any case of suspected pancreatitis or unexplained abdominal pain [**20**]. A contrast-enhanced CT scan at admission showed a high-dimensioned pancreas, particularly at the head, with heterogeneous structure and peripancreatic collections (**Fig. 2**). We scored the results according to the following computed tomography index: grade A – normal appearance of the pancreas (0 points); grade B – pancreatic enlargement (1 point); grade C – peripancreatic inflammation or gland abnormalities (2 points); grade D – single fluid collection (3 points); and grade E – 2 or more fluid collections or adjacent gas bubbles (4 points) [**2**]. Using this scale, we assigned a score of 4 points to our patient. Still, a CT scan is far more accurate than an abdominal ultrasound in delineating peripancreatic inflammation and detecting intrapancreatic necrosis [**14**]. The laboratory findings at admission showed high values for total lipids = 2600 mg/dL, triglycerides 1200 mg/dL and serum amylase of 86, 04 mg/dL. In most reported cases of pancreatitis, liver enzymes, bilirubin, and amylase are either normal or slightly elevated [**21**]. While pancreatitis rarely occurs except when triglyceride levels exceed 1500 mg/dL [**22**], the detection of mild to moderately elevated levels of triglycerides (200-1000 mg/dL) usually occurs in the early stages of AP of any etiology [**23**]. Janowitz considered serum amylase to be the most important diagnostic aid in determining pancreatic injury [**24**]. Although serum amylase may be normal in pancreatitis [**25**], up to 95% of cases of acute pancreatitis have elevated amylase levels [**26**]. The management of our patient’s pancreatitis was adopted according to the particularities of the case. As such, the patient was treated by fasting, gastric decompression by nasogastric tube, and intravenous antibiotherapy, followed by antialgic treatment and omega-3 fatty acids. The standard treatment of pancreatitis consists of bowel rest and IV fluids, with or without nasogastric suction. In 30% to 76% of cases, pediatric pancreatitis can be treated conservatively [**15**]. Isolated cases of AP due to hypertriglyceridemia have been reported and treated with plasma and lipoprotein apheresis with favorable outcomes. Ocreotide, a somatostatin analogue, has also been used because it activates receptors in the pancreas that modulate pancreatic exocrine secretion [**12**]. The mortality in pediatric pancreatitis varies greatly (0% - 78%) [**27**]. Nearly a quarter of children with acute pancreatitis develop a severe complication, and the mortality rate in these instances is of approximately 4%, despite significant advances in the treatment of this disease [**28**]. In conclusion, we reported a new case of acute pancreatitis secondary to hyperlipidemia and reviewed the pediatric literature on this disorder. Amidst the potential for misdiagnosis, we confirmed the idea that abdominal US should be performed in any case of suspected pancreatitis or unexplained abdominal pain. Thus, while patients with hyperlipidemia are prone to recurrent pancreatitis, the prognosis for pediatric patients with pancreatitis is good [**29**].
